# Impact of Body Mass Index on Local Recurrence according to Intrinsic Subtype Approximation in Korean Women with Early Stage Invasive Breast Cancer Receiving Contemporary Treatments

**DOI:** 10.7150/jca.59064

**Published:** 2021-06-01

**Authors:** Sang-Won Kim, Mison Chun, Yong Sik Jung, Young-Taek Oh, O Kyu Noh, Oyeon Cho

**Affiliations:** 1 Department of Radiation Oncology, Konyang University College of Medicine, Daejeon, Republic of Korea.; 2 Myunggok Medical Research Institute, Konyang University College of Medicine, Daejeon, Republic of Korea.; 3 Department of Radiation Oncology, Ajou University School of Medicine, Suwon, Republic of Korea.; 4 Department of Surgery, Ajou University School of Medicine, Suwon, Republic of Korea.

**Keywords:** breast cancer, body mass index, intrinsic subtype, local recurrence, prognostic factor

## Abstract

**Purpose:** We investigated the prognostic impact of body mass index (BMI) on local recurrence (LR) according to intrinsic subtype in Korean women with early stage, invasive breast cancer.

**Materials and methods:** We included 907 patients with pathological stage T1-2 and N0-1 breast cancer who underwent curative surgery between 2007 and 2012. Systemic treatments were administered in 876 patients (96.6%). In total, 701 patients (77.3%) received radiotherapy. Intrinsic subtypes were determined using immunohistochemical staining results.

**Results:** During the median follow-up period of 72 months, LR as the first failure occurred in 29 patients, including 24 patients with isolated LR. The 5-year cumulative incidence rate of LR was 3.2% among all patients. In the luminal A subtype, a BMI of <18.5 kg/m^2^ was an independent risk factor for LR, as determined by a competing-risk regression model (relative risk, 3.33; *p* = 0.041). Severely obese patients (BMI >30 kg/m^2^) with the triple negative subtype had an increased risk of LR (relative risk, 3.81; *p* = 0.048).

**Conclusion:** The present study identified traditionally underestimated risk groups for LR. BMI may diversely influence the rate of LR across intrinsic subtypes in Korean patients with breast cancer.

## Introduction

The steadily increasing prevalence of obesity is an important global health issue. The World Health Organization defines overweight and obesity as abnormal or excessive fat accumulation that presents a risk to health, and has referred to obesity as a disease since 1997 1. Recently, the American Medical Association also officially declared obesity as a chronic disease. This reflects the severity of obesity with regard to its consequences and associated socioeconomic burden.

The degree of obesity is usually expressed using body mass index (BMI), which is calculated as weight in kilograms divided by the square of height in meters. High BMI is an established major risk factor for various chronic diseases, such as hypertension, diabetes mellitus, and other metabolic syndromes. Along with these comorbidities, accumulating evidence describes the increased development of several types of malignancy in patient cohorts with a higher BMI 2-4. For example, a 1 unit increase in BMI increases the risk of breast cancer incidence by 1.07 times in postmenopausal Korean women 3.

BMI affects many aspects of breast cancer, including cancer occurrence, the determination of intrinsic subtypes, and prognosis 3-10. With regard to prognosis, three meta-analyses have demonstrated the association of high BMI with poor prognosis of breast cancer 7-9. However, there have been limited studies addressing the association of BMI with local recurrence (LR) and the results were inconsistent among these studies 11-16.

The prognostic impact of BMI is relatively consistent in hormone receptor-positive breast cancer and less consistent in hormone receptor-negative breast cancer 17, 18. This finding implies different levels of risk for LR associated with BMI, according to the hormone receptor status or intrinsic subtype; however, previous studies have not investigated the relationship between BMI and LR according to hormone receptor status or intrinsic subtype.

In addition to being overweight and/or obese, several researchers showed an increased risk of LR and mortality from breast cancer in patients who are underweight 11, 13, 19. However, owing to its rare incidence, the effects of being underweight have not received as much interest as those of obesity. As increased mortality from cancer in underweight patients has been observed mostly in East Asian populations 13, 19, 20, clinicians from these countries should investigate the role of being underweight as an important prognostic factor for cancer treatment.

As even isolated LR can increase breast cancer-specific mortality 21, 22, identifying high-risk groups for LR is important for decreasing the risk of death from cancer. The present study aimed to examine the association between BMI and LR according to each intrinsic subtype in Korean patients with breast cancer who received curative treatments based on the contemporary guidelines.

## Materials and methods

The Institutional Review Board of Ajou University School of Medicine approved this retrospective study with an exemption of informed consent. All patients with pathologically stage T1-2 and N0-1 who underwent curative surgery between 2007 and 2012 were included in the present study (*n* = 1,016). Patients who received preoperative treatments (*n* = 81), who had a history of malignancies (except papillary thyroid cancer) (*n* = 14), who presented with bilateral invasive breast cancer (*n* = 7), and who were lost to follow-up without any examinations for their treatment response (*n* = 7) were excluded.

The remaining 907 patients were diagnosed with pathological stage T1-2 and N0-1 tumors. In total, 721 patients underwent breast-conserving surgery and 186 underwent total mastectomy.

With the exception of 8 patients, all patients received adjuvant treatments. Of these, systemic treatments were administered in 878 patients: adjuvant chemotherapy alone in 196 patients, adjuvant hormone therapy alone in 234 patients, and both treatments in 448 patients. Patients with node-positive breast cancer received a taxane-based regimen. Patients with node-negative breast cancer received either 6 cycles of cyclophosphamide, methotrexate, and fluorouracil or 4-6 cycles of anthracycline and cyclophosphamide with or without fluorouracil. The chemotherapy dose was not reduced owing to BMI. Adjuvant trastuzumab was administered in 64 out of 135 patients who were human epidermal growth factor receptor 2 (HER2)-positive because of the approval of its adjuvant use by Korean National Health Insurance subsequent to the year 2007, or patient economic problems.

Radiotherapy was delivered in 701 patients. Twenty two patients did not receive radiotherapy after breast conserving surgery because of old age (*n* = 11) or their preference (*n* = 11). Although our institutional policy was to recommend against postmastectomy radiotherapy in N1 disease at the time of study, 2 patients received postmastectomy radiotherapy at their preference. The whole breast was irradiated conventionally with a total dose of 45-50.4 Gy and the tumor bed was boosted with 10-14 Gy. Thirty-two patients with multiple positive lymph nodes received supraclavicular irradiation with a total dose of 45 Gy.

In this study, we used cut-off points of BMI based on Asian criteria because of study population consisting of Korean women only 23. The cut-off points used in this study were as follows: <18.5 kg/m^2^ (underweight); 18.5-22.9 kg/m^2^ (normal); 23-24.9 kg/m^2^ (overweight); 25-29.9 kg/m^2^ (moderate obese); and ≥30 kg/m^2^ (severe obese).

Patients were approximated into 4 intrinsic subtypes based on the receptor status, detected by immunohistochemical staining as follows: luminal A (estrogen receptor [ER] or progesterone receptor [PR] positive, and HER2 negative), luminal B (ER or PR positive, and HER2 positive or Ki-67 index ≥14%), HER2-enriched (ER and PR negative, HER2 positive) and triple negative (ER, PR, and HER2 negative). Tumors were considered HER2 positive only if they were scored 3+ by immunohistochemistry or scored 2+ with HER2 gene amplified by fluorescence or silver *in situ* hybridization.

### Statistical analysis

Descriptive statistics were analyzed to compare the categorical variables of clinical, disease and treatment-related factors among intrinsic subtypes and by BMI using chi-squared or Fisher's exact tests. Continuous variables including age and BMI were compared using the Mann-Whitney U test. Follow-up was determined from the date of initial surgery. LR was defined as the first tumor recurrence in the ipsilateral breast or chest wall, with or without any other recurrences. All suspicious LRs on imaging work-ups were confirmed using needle or excisional biopsy. The LR rate was estimated using cumulative incidence analysis as described by Gray 24, and the competing risks were regional recurrence at axillary, internal mammary and/or supraclavicular lymph nodes area, distant metastasis, contralateral breast cancer, or death from any causes. A competing risk regression model was used to identify risk factors for LR, and variables with a *p* value <0.20 in the univariate regression model were incorporated into the multivariate analysis. BMI was included in the regression model after dichotomization according to the reference value of Asian criteria 23. Statistical analyses were performed using R software version 3.2.5 (https://cran.r-project.org/).

## Results

Patient characteristics are summarized in Table 1. The median BMI in all patients was 23.2 kg/m^2^ (range 15.4-36.5 kg/m^2^). The median BMI was not significantly different among intrinsic subtypes. Patients with the triple negative subtype showed the highest percentage of pathological stage T2 tumors. A higher percentage of patients with HER2-positive tumors received mastectomy. Adjuvant chemotherapy was administered more frequently to patients with hormone receptor-negative tumors.

The median age of all patients was 53 years (range, 19-85 years) and patients with BMI <18.5 kg/m^2^ were younger (median, 32 years) than other BMI groups (median, 50 years). A close (<2 mm) or positive final resection margin (the presence of tumor cells on the inked surface of the surgical specimen) was more common in women with a BMI <18.5 kg/m^2^ (40.7% in BMI <18.5 kg/m^2^ vs. 17.0% in BMI ≥18.5 kg/m^2^, *p* = 0.004).

The median follow-up period was 72 months (range 6-118 months) for all patients. A total of 82 patients experienced treatment failure. LR with or without other recurrence as the first failure occurred in 29 patients, including 24 patients with isolated LR. Fifty-three patients experienced regional recurrence (*n* = 21), distant metastasis (*n* = 32), and/or contralateral breast cancer (*n* = 9) as the first failure.

**Figure 1:**
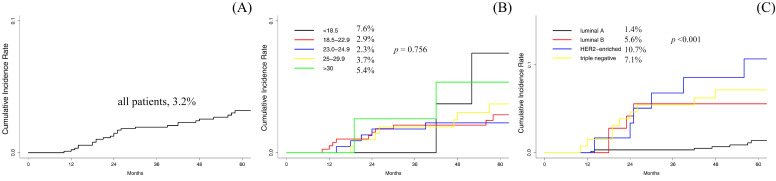
Plots of the 5-year cumulative incidence of local recurrence. (A) all patients, (B) among each BMI range and (C) among each approximated intrinsic subtype of breast cancer.

The 5-year cumulative incidence of LR was 3.2% (95% confidence interval [CI], 2.1-4.6%) for all patients (Fig. 1A). BMI was not significantly associated with LR in all patients (Fig. 1B). Age <35 years, final margin status, histologic grade and intrinsic subtypes had a significantly higher incidence of LR (Table 2). Luminal A had the lowest 5-year cumulative incidence of LR (1.4%) among all intrinsic subtypes (Fig. 1C). After multivariate competing risk regression, an age <35 years (relative risk [RR], 2.92; 95% CI, 1.225-6.96; *p* = 0.016), close or positive resection margin (RR, 4.28; 95% CI, 1.985-9.21; *p* <0.001) and intrinsic subtype (RR, 1.65; 95% CI, 1.256-2.17; *p* <0.001) were independent risk factors for LR in all patients.

**Figure 2:**
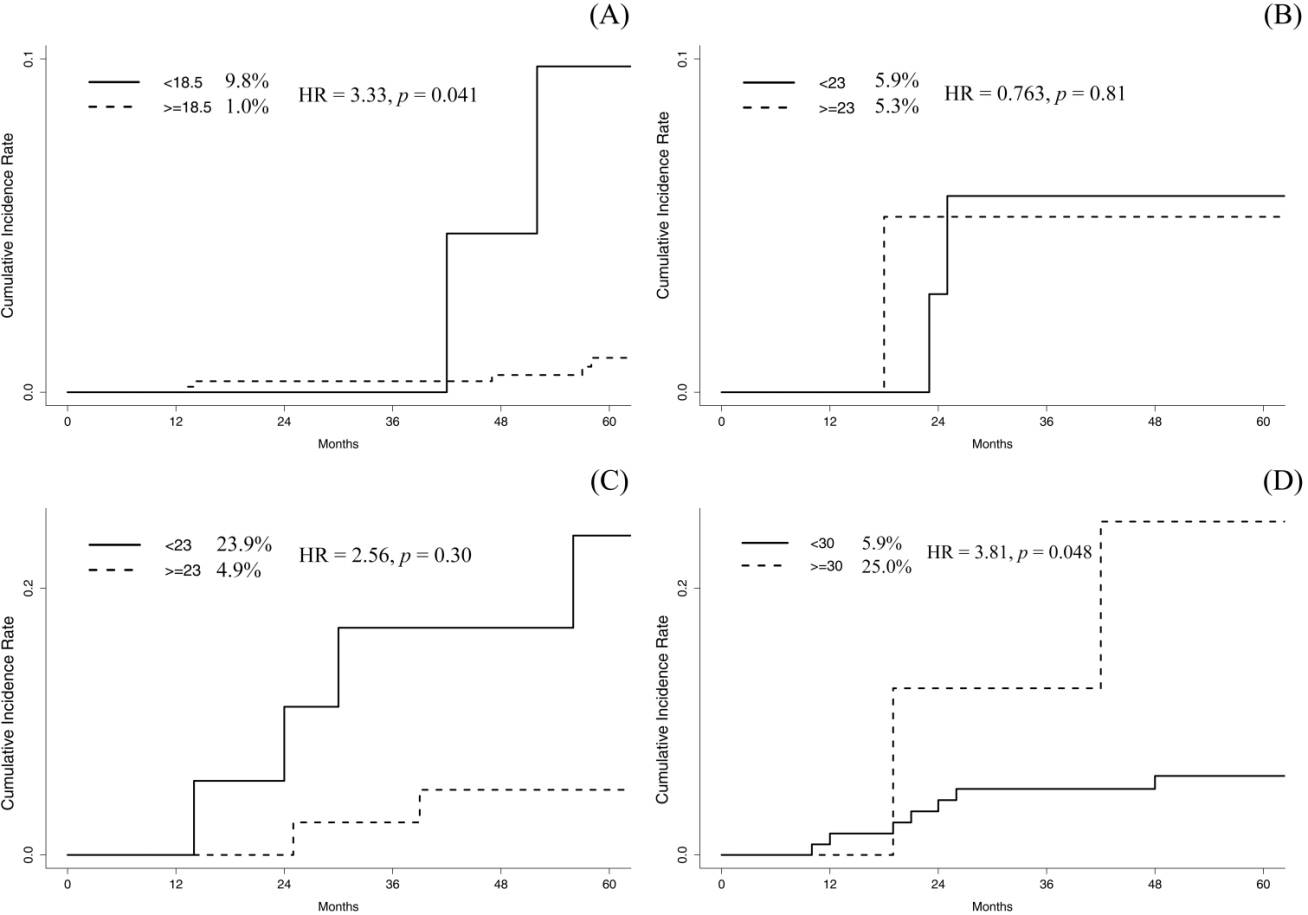
Plots of the 5-year cumulative incidence of local recurrence. (A) comparison between BMI <18.5 kg/m^2^ and >18.5 kg/m^2^ in luminal A subtype, (B) comparison between BMI <23 kg/m^2^ and >23 kg/m^2^ in luminal B subtype, (C) comparison between BMI <23 kg/m^2^ and >23 kg/m^2^ in HER2-enriched subtype and (D) comparison between BMI <30 kg/m^2^ and >30 kg/m^2^ in triple negative subtype.

The prognostic impact of BMI was evaluated according to each intrinsic subtype. In luminal A subtype, patients with a BMI <18.5 kg/m^2^ had a significantly increased risk of LR (9.8% in BMI <18.5 kg/m^2^ vs. 1.0% in BMI ≥18.5 kg/m^2^, *p* = 0.005; Fig. 2A). Multivariate competing risk regression model revealed that a BMI <18.5 kg/m^2^ was an independent prognostic factor for LR (RR, 3.33; 95% CI, 1.048-10.55; *p* = 0.041). An age <35 years (RR, 3.12; 95% CI, 1.018-10.80; *p* = 0.047) and close or positive resection margin (RR, 7.48; 95% CI, 2.010-27.83; *p* = 0.003) were also significantly associated with LR in the luminal A subtype. In the luminal B subtype, no variables were significantly associated with the cumulative incidence of LR (Fig. 2B).

In the HER2-enriched subtype, patients with a BMI <23 kg/m^2^ tended towards an increased cumulative incidence of LR (*p* = 0.086; Fig. 2C). In the multivariate regression analysis, a BMI <23 kg/m^2^ was not significantly associated with LR (RR, 2.56; 95% CI, 0.431-15.17; *p* = 0.30). Patients with a BMI ≥30 kg/m^2^ had a significantly increased incidence of LR in triple negative subtype (*p* = 0.046; Fig. 2D). After multivariate regression analysis, a BMI ≥30 kg/m^2^ remained as the only significant risk factor for LR (RR, 3.81; 95% CI, 1.012-14.37; *p* = 0.048).

## Discussion

The association between high BMI and an increased risk of LR has been inconsistent among certain studies 11-16. In general, large scale studies reported that BMI had no influence on LR 12, 14, while single institutional studies reported that BMI was significantly associated with LR 11, 15, 16. Patients from large-scale studies were included for a long period, and thereby consisted of a heterogeneous group receiving different diagnostic and therapeutic modalities 12, 14. Although single institutional studies had limited numbers of patients, patients consisted of homogeneous populations and received reasonably uniform treatments during the relatively short study period 11, 15, 16.

In contrast to the prevailing view that a higher BMI is significantly associated with LR, several studies have shown that lower BMI may increase the risk of LR. Marret et al. reported that a 1 unit elevation of BMI reduced the risk of LR approximately by 8% 11. Moon et al. also reported that being underweight was an independent prognostic factor for LR in 24,698 Korean women with invasive breast cancer 13. Although the association between BMI and hormone receptor status was not documented in the study by Moon et al., the present study further demonstrated that the detrimental effect of being underweight on LR was observed in the luminal A subtype. Kawai et al. also reported that being underweight significantly increased the risk of mortality from breast cancer in Japanese patients who were hormone receptor-positive 10.

The reason for the increased incidence of LR in patients who are underweight has not been comprehensively elucidated. Marret et al. speculated that large breast size in obese patients might be responsible for the delayed detection of LR 11. These authors also raised the possibility of a suboptimal resection margin, although this information was not available for their study. In addition, Moon et al. suggested that the poor prognosis of patients who were underweight might be attributable to a younger median age or possible malnutrition 13.

In the present study, patients who were underweight tended to have two major risk factors for LR in common: young age and suboptimal final margin status. Therefore, these factors might confound the results of the present study. A suboptimal final margin status was significantly more common in underweight patients than those who had a normal weight, were overweight or obese. Furthermore, a suboptimal final margin status was a strong prognostic factor for LR in all patients and this effect was more notable in the luminal A subtype. Presumably, a close relationship between being underweight and having a suboptimal final margin status complicates the effect of being underweight on LR.

The negative effects of young age on the outcomes of breast cancer, including LR, were more prominent in Korean patients who were hormone receptor-positive than those who were hormone receptor-negative 25, 26. In the present study, the increased incidence of LR in patients who were underweight was only observed in the luminal A subtype. Given the young median age in patients who were underweight, it could be assumed that the significantly higher incidence of LR in underweight patients within the luminal A subtype was attributable to the effect of young age. The present study showed that underweight remained as an independent risk factor after multivariate regression analysis adjusting for age and final margin status in luminal A subtype. However, it was challenging to completely rule out the connection between being underweight with a young age or the final margin status. Examining the prognostic effect of BMI in young age cancer patients is required to control this potential confounding factor. The present study could not investigate the prognostic effect of underweight in young patients because of limited patient numbers. Furthermore, the effects of being underweight on LR in other subtypes could not be assessed because the majority of patients who were underweight presented with the luminal A subtype.

In the present study, triple negative breast cancer patients with BMI ≥30 kg/m^2^ composed another risk group for higher incidence of LR, in addition to underweight patients with the luminal A subtype. The influence of BMI on the survival outcomes of triple negative breast cancer has been controversial 27-31. However, a positive association in obese patients with the triple negative subtype was consistently reported by several studies in East Asian populations. Choi et al. reported that, among patients with triple negative breast cancer, those with a BMI ≥30 kg/m^2^ had significantly reduced disease-free survival 32. Bao et al. demonstrated that obesity (BMI ≥28 kg/m^2^) at pre-diagnosis was significantly associated with a higher risk of total mortality and reduced breast cancer-specific survival in Chinese patients with breast cancer 33. Another study from China involving 1,106 patients with triple negative breast cancer also demonstrated that being overweight was an independent risk factor for breast cancer-specific survival and overall survival in premenopausal women 34.

The present study had several limitations, including inherent biases due to the retrospective design. The number of patients in the present study was small, particularly considering modest effect size of BMI on the prognosis of patients with breast cancer. Adjuvant trastuzumab was not administered to all HER2-positive patients and the effect of BMI on HER2-positive patients could not be assessed accurately. The definition of obesity by BMI and the classification of intrinsic subtypes through immunohistochemical results were approximated. Because a substantial number of patients had no record of serial weights, the influence of weight change was not evaluated. Finally, the results of the present study cannot be applied equally to other populations as we included only Korean patients.

In conclusion, the present study demonstrated a distinct relationship between BMI and the risk of LR in Korean patients with breast cancer, which may be different compared with European or North American populations. Patients who were underweight in the luminal A subtype and those who were obese in the triple negative subtype which have not been typically recognized as a risk group, were highly susceptible to LR after contemporary treatments with a curative aim in Korean patients with breast cancer. Therefore, the prognostic effects of BMI on breast cancer should be investigated with a multimodal approach based on genetic diversity across ethnic groups. In addition, tailored treatment guidelines for breast cancer, considering the BMI, age, and genetic background should be discussed.

## Figures and Tables

**Table 1:** Patient characteristics according to 4 intrinsic subtypes

Variables	Luminal A (n =636)	Luminal B (n = 73)	HER2-enriched (n = 62)	Triple negative (n = 136)	*p* values
age (median)	47	47	52	48	0.773
**Menstruation**					0.021
premenopausal	403 (63.4)	42 (57.5)	28 (45.2)	76 (55.9)	
postmenopausal	233 (36.6)	31 (42.5)	34 (54.8)	60 (44.1)	
**Childbirth**					0.045
nulliparous	61 (9.6)	6 (8.2)	2 (3.2)	21 (15.4)	
parous	575 (90.4)	67 (91.8)	60 (96.8)	115 (84.6)	
BMI (median)	23 kg/m^2^	23.4kg/m^2^	24.2 kg/m^2^	23.4 kg/m^2^	0.409
**Laterality**					0.125
left	311 (48.9)	38 (52.1)	37 (59.7)	79 (58.1)	
right	325 (51.1)	35 (47.9)	25 (40.3)	57 (41.9)	
**Location**					0.275
outer	464 (73.0)	56 (76.7)	52 (83.9)	99 (72.8)	
inner	172 (27.0)	17 (23.3)	10 (16.1)	37 (27.2)	
**Operation**					<0.001
breast conserving	515 (81.0)	47 (64.4)	37 (59.7)	122 (89.7)	
mastectomy	121 (19.0)	26 (35.6)	14 (40.3)	14 (10.3)	
**FMS**					0.150
≥2 mm	515 (81.0)	67 (91.8)	51 (82.3)	113 (83.1)	
<2 mm	121 (19.0)	6 (8.2)	11 (17.7)	23 (16.9)	
**HG**					<0.001
1	156 (24.5)	2 (2.7)	1 (1.6)	0	
2	277 (43.6)	18 (24.7)	8 (12.9)	14 (10.3)	
3	188 (29.6)	53 (72.6)	52 (83.9)	119 (87.5)	
unknown	15 (2.4)	0	1 (1.6)		
**T stage**					0.002
1	454 (71.4)	44 (60.3)	45 (72.6)	76 (55.9)	
2	182 (28.6)	29 (39.7)	17 (27.4)	60 (44.1)	
**N stage**					0.023
0	498 (78.3)	49 (67.1)	45 (72.6)	115 (84.6)	
1	138 (21.7)	24 (32.9)	17 (27.4)	21 (15.4)	
**AJCC stage**					0.011
I	379 (59.6)	32 (43.8)	31 (50.0)	67 (49.3)	
II	257 (40.4)	41 (56.2)	31 (50.0)	69 (50.7)	
**Chemotherapy**					<0.001
no	223 (35.1)	19 (26.0)	11 (17.7)	10 (7.4)	
yes	413 (64.9)	54 (74.0)	51 (82.3)	126 (92.6)	
**Radiotherapy**					<0.001
no	137 (21.5)	27 (37.0)	25 (40.3)	17 (12.5)	
breast only	475 (74.7)	43 (58.9)	36 (58.1)	115 (84.6)	
breast + SCL	24 (3.8)	3 (4.1)	1 (1.6)	4 (2.9)	

HER2, human epidermal growth factor receptor type 2; BMI, body mass index; FMS, final margin status; HG, histologic grade; LVSI, lymphovascular space invasion; AJCC, American Joint on Cancer Committee; SCL, supraclavicular lymph node.

**Table 2:** Cumulative incidence rate of local recurrence in entire patients

Variables	%	
Overall	3.2	
**Age**		0.003
<35 years	8.7	
≥35 years	2.8	
**Menstruation**		0.414
premenopausal	3.4	
postmenopausal	2.9	
**BMI**		0.756
<18.5 kg/m^2^	7.6	
18.5-22.9 kg/m^2^	2.9	
23-24.9 kg/m^2^	2.3	
25-29.9 kg/m^2^	3.7	
≥30 kg/m^2^	5.4	
**Laterality**		0.688
left	3.4	
right	3.0	
**Location**		0.804
outer	3.0	
inner	3.8	
**Operation**		0.162
breast conserving	3.5	
mastectomy	1.9	
**FMS**		<0.001
≥2 mm	2.2	
<2 mm	8.7	
**HG**		0.014
1	0.9	
2	2.7	
3	4.6	
**Hormone receptor**		<0.001
ER and/or PR+	1.8	
ER- and PR-	8.3	
**HER2**		<0.001
negative	2.4	
overexpressed	7.9	
**Subtypes**		<0.001
luminal A	1.4	
luminal B	5. 6	
HER2-enriched	10.7	
triple negative	7.1	
**T stage**		0.528
1	3.0	
2	3.7	
**N stage**		0.513
0	3.4	
1	2.5	
**AJCC stage**		0.943
I	3.1	
II	3.3	
**Chemotherapy**		0.723
no	3.2	
yes	3.2	
**Radiotherapy**		0.513
no	2.3	
breast only	3.6	
breast + SCL	0	

BMI, body mass index; FMS, final margin status; HG, histologic grade; LVSI, lymphovascular space invasion; ER, estrogen receptor; PR, progesterone receptor; HER2, human epidermal growth factor receptor type 2; AJCC, American Joint on Cancer Committee; SCL, supraclavicular lymph node.
